# Correction for Participation Bias in Nonprobability Samples Using Multiple Reference Surveys

**DOI:** 10.1002/sim.70403

**Published:** 2026-02-23

**Authors:** Victoria Landsman, Lingxiao Wang, Ivan Carrillo‐Garcia, Aya A. Mitani, Peter M. Smith, Barry I. Graubard, Trang Bui, Nancy Carnide

**Affiliations:** ^1^ Institute for Work and Health Toronto Canada; ^2^ Division of Biostatistics, Dalla Lana School of Public Health University of Toronto Toronto Canada; ^3^ Department of Statistics University of Virginia Charlottesville Virginia USA; ^4^ Economic Statistics Methods Division Statistics Canada Ottawa Canada; ^5^ Division of Epidemiology, Dalla Lana School of Public Health University of Toronto Toronto Canada; ^6^ Biostatistics Branch, Division of Cancer Epidemiology and Genetics National Cancer Institute, National Institutes of Health Bethesda Maryland USA; ^7^ Department of Statistics and Actuarial Science University of Waterloo Waterloo Canada

**Keywords:** calibration, finite population inference, pseudo‐weights, raking ratio, variance estimation

## Abstract

Health researchers are increasingly adopting nonprobability sampling strategies in survey studies. However, the participation mechanism in such samples is unknown and estimated target parameters and exposure‐outcome associations obtained from nonprobability samples can be biased. Current approaches developed to support statistical inference from nonprobability samples are unable to accommodate more than one reference sample. In this paper, we propose a general framework to address participation bias in nonprobability samples using multiple reference surveys. Previously published methods that use one reference survey are special cases within this framework. We focus primarily on the calibration estimators, another important special case in the proposed framework. These estimators have greater flexibility in situations with limited access to survey microdata and are straightforward for practical implementation. We describe two methods for variance estimation that account for all sources of variability of the proposed estimators: (1) the Taylor linearization method, which provides an analytic formula for the variance estimator, and (2) the leave‐one‐out jackknife method, a replication estimator. We assess the performance of the various methods through an extensive simulation study, which demonstrated satisfactory performance of the raking ratio calibration estimator in situations with highly dispersed participation probabilities in nonprobability samples and markedly smaller variance estimates for continuous outcomes. Finally, we illustrate the application of these methods using data from a real‐world study of working adults in Canada.

## 1 Introduction

Large‐scale probability surveys are considered the gold standard in applied health research. These surveys are characterized by a rigorous sampling design that controls the selection mechanism of survey participants. More specifically, each participant is assigned a survey sampling weight to reflect their probability of selection (and thus the number of people in the target population they represent), allowing findings from the sample to be generalized to the target population. However, assembling such survey samples can be challenging due to the high cost of data collection, declining response rates, and the time required to develop accurate sampling frames from which potential participants can be sampled and recruited [1, 2]. To overcome these barriers, health researchers are increasingly adopting nonprobability sampling strategies and recruiting participants via in‐person settings (e.g., clinics, community centers), smartphone apps, social media platforms, membership lists, or opt‐in panels composed of individuals who have agreed to participate in surveys periodically. In contrast to large‐scale probability surveys, the participation mechanism in nonprobability samples is unknown. As such, nonprobability samples can often over‐ or under‐represent certain demographic, lifestyle, occupational, and health‐related characteristics in the target population, which can, in turn, lead to biased estimates of the target parameters. In addition, when factors associated with the outcomes and exposures of interest are associated with the decision of individuals to participate in the nonprobability sample, exposure‐outcome regression analyses can produce biased estimates of association parameters.

Recently, methods have been developed to support statistical inference from nonprobability samples, including the adjusted logistic propensity method [3] and the method proposed by Chen, Li and Wu [4] (see Sections 3.1 and 3.2 in that paper); hereafter called the ALP and CLW methods, respectively. The main premise behind these methods is to estimate an individual's probability to participate in a nonprobability sample using information from an external probability reference survey. The inverse of this estimated probability is a so‐called pseudo‐weight, similar to the survey sampling weight assigned to each individual in a probability survey. Specifying the participation model is the key step in the implementation of the ALP and CLW methods. All auxiliary variables that are potentially related to an individual's decision to participate in the nonprobability sample and are available in both the nonprobability sample and the reference survey should be harmonized to be included in the participation model.

Both ALP and CLW methods have been developed for a single reference survey that is assumed to contain all the auxiliary variables associated with participation in the nonprobability sample. However, in many practical situations, a single reference sample will lack some potentially important auxiliary variables. This was the case with the cannabis study sample discussed in Section 7, in which working‐age adults were interviewed about their cannabis use and perceptions of cannabis use at work. It is plausible to assume that variables related to both occupational and health characteristics may have determined an individual's decision to participate in this type of survey. However, no single available reference survey contained the information about both work and health required for specification of the participation model. Omitting important auxiliary variables from the participation model will lead to bias in the subsequent pseudo‐weighted analyses using the data from the nonprobability sample. While additional reference sample(s) may contain the potentially important missing auxiliary variables, currently available approaches are unable to accommodate more than one reference sample in the generation of pseudo‐weights. In this paper, we address this gap by describing a general statistical framework that allows for the integration of multiple reference samples to generate pseudo‐weights for nonprobability samples. This framework also includes the ALP and CLW methods for one reference survey as special cases.

The remainder of the paper is organized as follows. In Section 2, we define the problem, introduce the notation, and state the assumptions. We describe the general framework for estimation of pseudo‐weights and, consequently, the population means using one reference survey in Section 3. We also show that the ALP and CLW methods are special cases within this framework and describe the calibration estimator. In Section 4, we propose to extend this general framework to include multiple reference surveys, focusing our attention on the calibration estimator. Section 5 addresses variance estimation. In particular, we use the Taylor linearization method to derive analytic formulae for variance estimators and describe a leave‐one‐out jackknife variance estimator. In Section 6, we design and implement a simulation study to evaluate the performance of the different pseudo‐weighted estimators discussed in Sections 3 and 4. We then illustrate the application of these estimators in a real‐world example in Section 7. We conclude with a brief discussion in Section 8.

## 2 Problem Statement

Let FP denote the finite target population of size N and $\left\{y_{i},x_{i}\right\}$ denote the values of the outcome variable y and a set of L auxiliary variables $x_{i}=(x_{i1},\cdots \;,x_{iL})$ for each i∈FP (recorded without measurement errors). The finite population mean $\mu =\sum_{i\in \text{FP}}y_{i}/N$ is the target parameter in this study.

Suppose a nonprobability sample $s_{c}$ of size $n_{c}$ is obtained from FP and define $\delta_{i}^{c}$ to be the indicator variable that takes the value of 1 if i∈FP is included in the nonprobability sample $s_{c}$ and the value of 0 otherwise. We start with the positivity assumption about $\pi_{i}^{c}:=\mathbb{P}(\delta_{i}^{c}=1|i\in \text{FP})$, which is referred to as a participation probability.

Assumption 1 Each unit in FP has a nonzero probability of being included in the nonprobability sample: $\pi_{i}^{c}>0$ for each i∈FP.

The two inverse probability weighted estimators commonly used to estimate μ are the Horvitz–Thompson [5] (H‐T) and Hajek [6] estimators:

(1) $${\hat{\mu }}_{\text{HT}}=N^{-1}\underset{i\in s_{c}}{\sum }\frac{y_{i}}{\pi_{i}^{c}}\;\text{and}\;{\hat{\mu }}_{\text{Hajek}}={\hat{N}}^{-1}\underset{i\in s_{c}}{\sum }\frac{y_{i}}{\pi_{i}^{c}}$$

where $\hat{N}=\sum_{i\in s_{c}}(1/\pi_{i}^{c})$. Both estimators are consistent for μ under Assumption 1 and standard regularity conditions if the true participation probabilities $\pi_{i}^{c}$ are known [7]. Here, we focus on the Hajek estimator (${\hat{\mu }}_{\text{Hajek}}$), as it has been shown to have better efficiency and is recommended even when N is known [8]. For brevity, we omit the subscript ‘Hajek’ and define $\hat{\mu }:={\hat{\mu }}_{\text{Hajek}}$.

The participation probabilities $\pi_{i}^{c}$ are generally unknown in the nonprobability sample and need to be estimated. Utilizing additional information from an available probability (reference) survey has been proposed to estimate these probabilities. For generality, assume that we have access to K≥1 probability surveys ${\left\{s_{p_{k}}\right\}}_{k=1}^{K}$ of sizes $n_{p_{1}},n_{p_{2}},\ldots ,n_{p_{K}}$, respectively. Each reference survey is selected from the same finite population FP with known probabilities $\pi_{i}^{\left(p_{k}\right)}:=\mathbb{P}(\delta_{i}^{\left(p_{k}\right)}=1|i\in \text{FP})$, where $\delta_{i}^{\left(p_{k}\right)}$ is the indicator variable that takes the value of 1 if i∈FP is selected in $s_{p_{k}}$ and the value of 0 otherwise, for k=1,…,K. We adopt the following assumptions about the selection processes of $s_{c}$ and $s_{p_{k}}$.

Assumption 2 For each i∈FP, the indicator variable $\delta_{i}^{c}$ and the response variable $y_{i}$ are independent given the set of L auxiliary variables in $x_{i}$; that is, $\pi_{i}^{c}=\mathbb{P}(\delta_{i}^{c}=1|y_{i},x_{i})=\mathbb{P}(\delta_{i}^{c}=1|x_{i})$ for i∈FP.

Assumption 3 The K≥1 reference surveys randomly selected from the same finite population FP with probabilities $\pi_{i}^{\left(p_{k}\right)}>0$ (k=1,…,K and i∈FP) collectively contain all L auxiliary variables in $x_{i}$ so that Assumption 2 holds.

Assumption 4 For each $i,i^{\prime }\in \text{FP}$, the indicator variables $\delta_{i}^{c}$ and $\delta_{i^{\prime }}^{c}$ are independent given $x_{i}$ and $x_{i^{\prime }}$ for $i\neq i^{\prime }$.

Assumption 5 For each i∈FP and 1≤k≠l≤K, the indicator variables $\delta_{i}^{c}$, $\delta_{i}^{\left(p_{k}\right)}$, and $\delta_{i}^{\left(p_{l}\right)}$ satisfy $\text{cov}(\delta_{i}^{c},\delta_{i}^{\left(p_{k}\right)})=0$ and $\text{cov}(\delta_{i}^{\left(p_{k}\right)},\delta_{i}^{\left(p_{l}\right)})=0$.

Assumptions 2, 3 reflect the noninformative participation in $s_{c}$ given the auxiliary variables x. The K reference surveys ${\left\{s_{p_{k}}\right\}}_{k=1}^{K}$ collectively contain these variables, which are also observed in the nonprobability sample $s_{c}$. Under Assumption 4, Poisson sampling is used to approximate the selection process of $s_{c}$ in all variance derivations in this paper. However, variance derivations can be written under other complex designs if this assumption is not feasible and needs to be relaxed. Assumption 5 allows for elegant variance decomposition between different sources of variation involved in estimating probabilities $\pi_{i}^{c}$. Importantly, the methods discussed in this paper are designed for situations in which outcomes of interest are measured only in the nonprobability sample and are not available in any of the reference surveys.

## 3 Estimation Using One Reference Survey

In this section, we assume that a single reference survey $s_{p}$ contains all the auxiliary variables so that Assumption 2 holds. We also assume a parametric model $\pi_{i}^{c}:=\pi^{c}(x_{i},\beta )$, where $\beta =(\beta_{0},\beta_{1},\ldots ,\beta_{L})$ is a vector of nuisance parameters and $x=(1,x_{1},\ldots ,x_{L})$ is a vector of size L+1. There can be multiple choices for the functional form of $\pi^{c}(x_{i},\beta )$ (see Section 3.1 for examples). When the parametric model of $\pi^{c}(x_{i},\beta )$ is correctly specified, β can be estimated approximately unbiasedly by solving a class of L+1 estimating equations

(2) $$N^{-1}\overset{N}{\underset{i=1}{\sum }}\delta_{i}^{c}w(x_{i},\beta )h(x_{i},\beta )-N^{-1}\overset{N}{\underset{i=1}{\sum }}\delta_{i}^{p}d_{i}h(x_{i},\beta )=0$$

where $h(x_{i},\beta )$ is a prespecified vector‐valued smooth function of the same dimension as β and $d_{i}=1/\pi_{i}^{p}$ are the (known) sampling weights for the reference survey $s_{p}$. The sample versions of these equations can be solved using a Newton–Raphson iterative algorithm, leading to a solution denoted by $\hat{\beta }$. Then, the resulting pseudo‐weighted Hajek estimator of the FP mean can be obtained by

(3) $$\hat{\mu }\left(\hat{\beta }\right)={\left\{\overset{N}{\underset{i=1}{\sum }}\delta_{i}^{c}w\left(x_{i},\hat{\beta }\right)\right\}}^{-1}\left\{\overset{N}{\underset{i=1}{\sum }}\delta_{i}^{c}w\left(x_{i},\hat{\beta }\right)y_{i}\right\}$$

Notice that although $h(x_{i},\beta )$ is arbitrary, the choice of $h(x_{i},\beta )$, combined with the functional form of $w(x_{i},\beta )$, can result in different values of $\hat{\beta }$ and $\hat{\mu }(\hat{\beta })$ with different efficiencies (see Section 6 for details). In the following, we describe two previously published pseudo‐weighting approaches as special cases of the general estimating equations approach above.

To make inference about the estimator $\hat{\mu }\left(\hat{\beta }\right)$ defined in (3), define $\eta ={\left(\mu ,\beta^{⊤}\right)}^{⊤}$ to be the vector of parameters. The estimator $\hat{\eta }={\left(\hat{\mu }(\hat{\beta }),\;{\hat{\beta }}^{⊤}\right)}^{⊤}$ combines $\hat{\mu }\left(\hat{\beta }\right)$ and the estimator $\hat{\beta }$ for the pseudo‐weights $w(x_{i},\beta )$, that can be obtained by solving the following system of the estimating equations:

(4) $$\begin{array}{ll} & \Phi (\eta ):=\left(\begin{array}{c} N^{-1}\overset{N}{\underset{i=1}{\sum }}\delta_{i}^{c}w(x_{i},\beta )(y_{i}-\mu )\\ N^{-1}\overset{N}{\underset{i=1}{\sum }}\delta_{i}^{c}w(x_{i},\beta )h(x_{i},\beta )-N^{-1}\overset{N}{\underset{i=1}{\sum }}\delta_{i}^{p}d_{i}h(x_{i},\beta ) \end{array}\right)=0 \end{array}$$

Under Assumptions 1, 2, 3, the estimating equations in (4) are unbiased in the sense that ${𝔼}_{cp}[\Phi (\eta_{0})]=0$, where $\eta_{0}$ is the true value of η and the expectation ${𝔼}_{cp}$ is taken with respect to the joint distribution of the indicator variables $\delta_{i}^{c}$ and $\delta_{i}^{p}$. The unbiasedness of the system of the estimating equations implies, under standard regularity conditions, the consistency of the estimator $\hat{\eta }$ using the theory of general estimating functions similar to those presented in Section 3.2 in Tsiatis [9], as well as in Binder [10] and Schiopu‐Kratina [11] for complex survey designs. Additionally, this theory allows derivation of the design‐based finite population variance of the estimator $\hat{\eta }$ as

(5) $${𝕍}_{cp}\left(\hat{\eta }\right)={\left\{{𝔼}_{cp}\left(\phi (\eta_{0})\right)\right\}}^{-1}{𝕍}_{cp}\left\{\Phi (\eta_{0})\right\}{\left\{{𝔼}_{cp}{\left(\phi (\eta_{0})\right)}^{⊤}\right\}}^{-1}$$

where ϕ(η)= ∂Φ(η)/∂η . See Appendix A for the derivation of the analytic expression for ${𝕍}_{cp}\left\{\hat{\mu }\left(\hat{\beta }\right)\right\}$.

### 3.1 Important Special Cases

#### 3.1.1 The ALP and CLW Estimators

The ALP [3] and CLW [4] methods proposed in the literature can be formulated as special cases of the general estimating equations approach described above. Specifically, using

(6) $$w(x_{i},\beta )=\exp(-\beta^{⊤}x_{i})\;\text{and}\;h(x_{i},\beta )={\left[1+w(x_{i},\beta )\right]}^{-1}x_{i}$$

in (2), implies the estimating equations presented in Section 2.3 of the ALP paper [3]; and using

(7) $$w(x_{i};\beta )=1+\exp(-\beta^{⊤}x_{i})\;\text{and}\;h(x_{i},\beta )={\left[w(x_{i},\beta )\right]}^{-1}x_{i}$$

in (2) implies the estimating equations presented in Section 3.1 of the CLW paper [4]. We denote the solutions of these estimating equations as ${\hat{\beta }}_{\text{ALP}}$ and ${\hat{\beta }}_{\text{CLW}}$, respectively.

Note that the ALP and CLW approaches assume different parametric forms of $\pi^{c}(x_{i},\beta )$, which, therefore, can lead to different values of $\hat{\beta }$, and, consequently, to the final estimates of $\hat{\mu }(\hat{\beta })$, especially when the sample fraction of the nonprobability sample (i.e., $f_{c}=n_{c}/N$) is large [3].

#### 3.1.2 The Calibration Estimators

Setting $h(x_{i},\beta )=x_{i}$ in (2) implies the estimating equations

(8) $$N^{-1}\overset{N}{\underset{i=1}{\sum }}\delta_{i}^{c}w(x_{i},\beta )x_{i}-N^{-1}\overset{N}{\underset{i=1}{\sum }}\delta_{i}^{p}d_{i}x_{i}=0$$

that resemble the calibration equations in Deville and Särndal [12]. In our setting, the pseudo‐weights $w(x_{i},\beta )$ for $i\in s_{c}$ are obtained by minimizing the averaged distance between the pseudo‐weights and the “naive” weights of 1 for all $i\in s_{c}$ under the condition (8). This condition ensures that the pseudo‐weighted totals of x from $s_{c}$ are equal to the survey‐weighted totals of x from $s_{p}$. The parametric form of the weight function $w(x_{i};\beta )$ is determined by the distance measure [12]. For example, the linear distance function is defined by $w(x_{i};\beta )=(1+\beta^{⊤}x_{i})$ and the multiplicative (also called raking ratio) distance function is defined by $w(x_{i};\beta )=\exp(-\beta^{⊤}x_{i})$. To avoid negative pseudo‐weights that can be produced by the linear distance, we choose the raking ratio distance function and denote the corresponding solution of (8) as ${\hat{\beta }}_{\text{cal}}$ and refer to the corresponding estimator of the mean as the raking ratio calibration estimator.

Notice that the raking ratio calibration and the ALP methods are defined by the same parametric form of the weight function $w(x_{i};\beta )=\exp(-\beta^{⊤}x_{i})$. Therefore, ${\hat{\beta }}_{\text{cal}}$ and ${\hat{\beta }}_{\text{ALP}}$ converge to the same true β if the weight function is correctly specified. However, ${\hat{\beta }}_{\text{cal}}$ and ${\hat{\beta }}_{\text{ALP}}$ can have different variances because the raking ratio calibration method uses $h(x_{i};\beta )=x_{i}$, whereas the ALP method uses $h(x_{i},\beta )={\left[1+w(x_{i},\beta )\right]}^{-1}x_{i}$. As a result, $\hat{\mu }({\hat{\beta }}_{\text{cal}})$ and $\hat{\mu }({\hat{\beta }}_{\text{ALP}})$ can have different efficiencies (see Sections 5 and 6 for theoretical and empirical results, respectively).

## 4 Estimation Using Multiple Reference Surveys

The calibration estimating equations in (8) can be extended further to the case where a single reference survey does not contain all the auxiliary variables that determine the individual's decision to participate in $s_{c}$. For that purpose, assume that we have access to K>1 probability reference surveys ${\left\{s_{p_{k}}\right\}}_{k=1}^{K}$ ($n_{p_{1}}<n_{p_{2}}<\ldots <n_{p_{K}}$) sampled from the same target population FP, which collectively contain all L auxiliary variables. Let $x^{\left(p_{k}\right)}$ define a vector of unique auxiliary variables available in both $s_{c}$ and $s_{p_{k}}$. If the surveys contain overlapping variables, these will be selected from the largest survey, without loss of generality, to ensure that each auxiliary variable appears in each vector $x^{\left(p_{k}\right)}$ (k=1,…,K) only once. Following this rule, a constant term ‘1’ that corresponds to the intercept $\beta_{0}$ in the participation model is included together with the auxiliary variables of the largest survey. For example, let K=2,L=3 and $n_{p_{2}}>n_{p_{1}}$. Assume that $\left\{x_{1},x_{2}\right\}$ are observed in $s_{p_{2}}$ and $\left\{x_{1},x_{3}\right\}$ are observed in $s_{p_{1}}$. Then, $x^{\left(p_{2}\right)}=(1,x_{1},x_{2})$ and $x^{\left(p_{1}\right)}=x_{3}$. In this example, the overlapping variable $x_{1}$ was selected from the reference survey $s_{p_{2}}$ because it is the largest survey.

Using this notation, the calibration estimating equations in (8) for one reference survey can be extended as

(9) $$\left\{\begin{array}{l} N^{-1}\overset{N}{\underset{i=1}{\sum }}\delta_{i}^{c}w(x_{i},\beta )x_{i}^{\left(p_{K}\right)}-N^{-1}\overset{N}{\underset{i=1}{\sum }}\delta_{i}^{\left(p_{K}\right)}d_{i}^{\left(p_{K}\right)}x_{i}^{\left(p_{K}\right)}=0\\ \;\;\;\;\;\;\ldots \\ N^{-1}\overset{N}{\underset{i=1}{\sum }}\delta_{i}^{c}w(x_{i},\beta )x_{i}^{\left(p_{1}\right)}-N^{-1}\overset{N}{\underset{i=1}{\sum }}\delta_{i}^{\left(p_{1}\right)}d_{i}^{\left(p_{1}\right)}x_{i}^{\left(p_{1}\right)}=0 \end{array}\right.$$

where $x={\left(x^{{\left(p_{K}\right)}^{⊤}},\cdots \;,x^{{\left(p_{2}\right)}^{⊤}},x^{{\left(p_{1}\right)}^{⊤}}\right)}^{⊤}$ is a vector of size L+1 and $d_{i}^{\left(p_{k}\right)}=1/\pi_{i}^{p_{k}}$, $i\in s_{p_{k}}$ are the (known) sampling weights in the reference survey $s_{p_{k}}$ for k=1,…,K.

Let ${\hat{\beta }}_{\text{cal}}$ be the solution to (9). The theoretical properties of the resulting Hajek estimator $\hat{\mu }\left({\hat{\beta }}_{\text{cal}}\right)$ follow from the unbiasedness of the estimating equations in (9) combined with the first equation in (4), similar to the case with one reference survey in Section 3. Using similar derivations to the case with one reference survey (see Appendix A), the analytic expression for the finite population variance ${𝕍}_{cp_{K}}\left\{\hat{\mu }\left({\hat{\beta }}_{\text{cal}}\right)\right\}$ can be obtained as

(10) $$\begin{array}{ll} & {𝕍}_{cp_{K}}\left\{\hat{\mu }\left({\hat{\beta }}_{\text{cal}}\right)\right\}\\ & \;=N^{-2}\overset{N}{\underset{i=1}{\sum }}\left\{w(x_{i},\beta )-1\right\}{\left(y_{i}-\mu -b^{⊤}x_{i}\right)}^{2}+N^{-2}b^{⊤}Db \end{array}$$

where $b^{⊤}=U_{\beta }S_{\beta }^{-1}$ with $U_{\beta }=N^{-1}\sum_{i=1}^{N}\pi^{c}(x_{i},\beta )(y_{i}-\mu ){\left(\frac{\partial w(x_{i},\beta )}{\partial \beta }\right)}^{⊤}$ and $S_{\beta }=N^{-1}\sum_{i=1}^{N}\pi^{c}(x_{i},\beta )x_{i}{\left(\frac{\partial w(x_{i},\beta )}{\partial \beta }\right)}^{⊤}$; and D is a block‐diagonal matrix with diagonal blocks defined by the matrices $D_{k}={𝕍}_{p_{k}}\left(\sum_{i=1}^{N}\delta_{i}^{\left(p_{k}\right)}d_{i}^{\left(p_{k}\right)}x_{i}^{\left(p_{k}\right)}\right)$. The variances ${𝕍}_{p_{k}}$ are determined according to the sampling design of the k‐th reference survey $s_{p_{k}}$.

The estimating equations in (9) require the estimated population totals of the auxiliary variables and the analytic variance formula (10) requires the corresponding variance estimates of these totals. These estimates can be obtained from survey microdata or from published resources provided by government agencies. This flexibility might be helpful in practical situations with limited access to survey microdata. The resulting calibration estimators also have a number of attractive features described in Section 6.

### 4.1 The Precalibration Procedure

The raking ratio calibration method proposed in Section 4 assumes that only unique variables are included in (9). However, certain auxiliary variables, particularly sociodemographic variables (e.g., age, sex, education) are routinely measured in probability surveys. In these situations, reference surveys will contain overlapping variables. To preserve the information on the relationships between the overlapping and the unique auxiliary variables within each reference survey, survey weights can be precalibrated such that the estimated weighted totals of the overlapping auxiliary variables, as well as the sum of the weights, are equal across the surveys. To illustrate this idea, consider the example above, in which $x_{1}$ is an overlapping variable in the two surveys. In this case, the overlapping variable $x_{1}$ will be selected from the larger reference survey ($s_{p_{2}}$). However, the weights of the smaller survey, $s_{p_{1}}$, can be precalibrated to ensure that the total of $x_{1}$ and the sum of the weights are equal to the corresponding totals in $s_{p_{2}}$. Since the variable $x_{1}$ in $s_{p_{1}}$ will not be used in (9), such precalibration procedure will ensure that the information on the relationship between $x_{1}$ and $x_{3}$ in $s_{p_{1}}$ is preserved in the precalibrated survey weights. In the case of no overlapping variables, the precalibration is performed only on the sum of the survey weights to ensure the same estimated size of the finite population. The precalibrated weights are further used in estimating equations in (9). In Section 6 we study the effect of the precalibration procedure on the bias and variance of the mean estimator under various simulated scenarios. The precalibration procedure requires access to the survey microdata and can be implemented using the function survey::calibrate [13] in R [14].

## 5 Variance Estimation

In this section, we describe two approaches for variance estimation: (1) the Taylor linearization approach to obtain the analytic formula for the variance estimator (Section 5.1) and (2) the leave‐one‐out jackknife approach, which is a replication approach (Section 5.2).

### 5.1 Analytic Formula Using Taylor Linearization Method

In the following, we derive an analytic formula for the variance estimator of the domain mean, which is also used in the real data application in Section 7. For this purpose, the domain mean estimator can be written as $\hat{\mu }:=\hat{\mu }(\hat{\beta })=\frac{T_{1}}{T_{2}}$, where $T_{1}=\sum_{i\in s_{c}}w(x_{i},\hat{\beta })y_{i}z_{i}$ and $T_{2}=\sum_{i\in s_{c}}w(x_{i},\hat{\beta })z_{i}$. Here, $z_{i}$ is an indicator variable that equals 1 for observations that are members of the domain (e.g., males) and zero, otherwise, and $w(x_{i},\hat{\beta })$ are the estimated pseudo‐weights. The overall mean estimator is a special case if $z_{i}=1$ for every $i\in s_{c}$.

By applying the Taylor deviates rules in Graubard and Fears [15] to any $i\in s_{c}\cup_{k=1}^{K}s_{p_{k}}$, the delta operator $\Delta_{i}(\hat{\mu })$ is computed as

(11) $$\Delta_{i}(\hat{\mu })=\frac{\partial \hat{\mu }}{\partial T_{1}}\Delta_{i}(T_{1})+\frac{\partial \hat{\mu }}{\partial T_{2}}\Delta_{i}(T_{2})=\frac{1}{T_{2}}[\Delta_{i}(T_{1})-\hat{\mu }\Delta_{i}(T_{2})]$$

where

$$\begin{array}{ll} \Delta_{i}(T_{1}) & =\Delta_{i}\left[\underset{j\in s_{c}}{\sum }w(x_{j},\hat{\beta })y_{j}z_{j}\right]\\ & =w(x_{i},\hat{\beta })y_{i}z_{i}\delta_{i}^{c}+\frac{\partial \left[\sum_{j\in s_{c}}w(x_{j},\hat{\beta })y_{j}z_{j}\right]}{\partial \hat{\beta }}\Delta_{i}(\hat{\beta }) \end{array}$$

and

$$\begin{array}{ll} \Delta_{i}(T_{2}) & =\Delta_{i}\left[\underset{j\in s_{c}}{\sum }w(x_{j},\hat{\beta })z_{j}\right]\\ & =w(x_{i},\hat{\beta })z_{i}\delta_{i}^{c}+\frac{\partial \left[\sum_{j\in s_{c}}w(x_{j},\hat{\beta })z_{j}\right]}{\partial \hat{\beta }}\Delta_{i}(\hat{\beta }) \end{array}$$

and the expression for $\Delta_{i}(\hat{\beta })$ is defined from the system of estimating equations depending on the method used to estimate $\hat{\beta }$ (see Appendix B for details in the case of the raking ratio calibration method).

The variance of the mean estimator $\hat{𝕍}\left(\hat{\mu }(\hat{\beta })\right)$ is then approximated by the variance of the sum of $\Delta_{i}(\hat{\mu })$, which, in turn, is determined by the sampling design of the corresponding surveys [15]. Each of the methods for estimating the pseudo‐weights mentioned above is characterized by different parametric forms for the functions w(·) and h(·). In Appendix B, we provide the derivation of $\Delta_{i}(\hat{\mu })$ for the raking ratio calibration estimator with one reference survey (i.e., $w(x_{i},\beta )=\exp(-\beta^{⊤}x_{i})$ and $h(x_{i},\beta )=x_{i}$) under the Assumptions 1, 2, 3, 4, 5. The extension to multiple reference surveys is straightforward and the derivations for other methods are very similar. Specifically, the estimated variance of the raking ratio calibration mean estimator with K reference surveys is given by

(12) $$\begin{array}{ll} & \hat{𝕍}\left(\hat{\mu }({\hat{\beta }}_{\text{cal}})\right)\\ & \;=T_{2}^{-2}\left[\underset{i\in s_{c}}{\sum }w(x_{i},\hat{\beta })[w(x_{i},\hat{\beta })-1]{\left(y_{i}z_{i}-\hat{\mu }z_{i}-{\hat{b}}^{⊤}x_{i}\right)}^{2}+{\hat{b}}^{⊤}\hat{D}\hat{b}\right] \end{array}$$

where ${\hat{b}}^{⊤}={\hat{U}}_{\beta }{\hat{S}}_{\beta }^{-1}=\left[\sum_{i\in s_{c}}w(x_{i},\hat{\beta })z_{i}(y_{i}-\hat{\mu })x_{i}^{⊤}\right]\left[\sum_{i\in s_{c}}w(x_{i},\hat{\beta })\right.{\left.x_{i}x_{i}^{⊤}\right]}^{-1}$ and $\hat{D}$ is a block‐diagonal matrix with diagonal blocks defined by the matrices ${\hat{D}}_{k}={\hat{𝕍}}_{p_{k}}\left(\sum_{i\in s_{p_{k}}}d_{i}^{\left(p_{k}\right)}x_{i}^{\left(p_{k}\right)}\right)$. The variances ${\hat{𝕍}}_{p_{k}}$ are estimated according to the sampling design of the k‐th reference survey $s_{p_{k}}$. The component of the estimated variance that corresponds to the nonprobability sample $s_{c}$ is obtained under the assumption of Poisson sampling.

### 5.2 Leave‐One‐Out Jackknife Estimator

A leave‐one‐out jackknife variance estimator can be used to approximate the variance estimator of the mean estimated from a multi‐stage stratified sample in which $k_{h}$ primary sampling units (PSUs) are sampled from a stratum h, h=1,…,H. The implementation of the method requires construction of $k_{h}\times L$ replicates of the estimated mean ${\hat{\mu }}_{\left(ht\right)}$ for $t=1,\ldots ,k_{h}$ in the following three steps: (1) exclude the observations of the t‐th PSU in the stratum h; (2) increase the sample weights of the remaining observations in the h‐th stratum by $k_{h}{\left(k_{h}-1\right)}^{-1}$ and do not change the weights in the other strata; (3) estimate the mean ${\hat{\mu }}_{\left(ht\right)}$ from the resulting replicate dataset. Then, the jackknife variance estimator is given by

(13) $${\hat{𝕍}}_{\mathrm{JK}}(\hat{\mu })=\overset{H}{\underset{h=1}{\sum }}\frac{k_{h}-1}{k_{h}}\overset{k_{h}}{\underset{t=1}{\sum }}{\left({\hat{\mu }}_{\left(ht\right)}-\hat{\mu }\right)}^{2}$$

where $\hat{\mu }$ is the mean estimator obtained from the entire data set. To implement the jackknife variance estimator in our setting, we view the combined sample $s_{c}\cup_{k=1}^{K}s_{p_{k}}$ as a stratified sample with H=K+1 strata, since all the samples are independently selected. The sampled individuals for $s_{c}$ are their own sampled PSU's so they are treated as a one‐stage sample. To create replicates in the stratum that corresponds to the nonprobability sample, the method of random groups [16] can be used. By this method, each random group plays the role of a sampled PSU, which is more computationally efficient than treating each individual as a sampled PSU.

## 6 Simulation Study

The simulation study design in this paper was inspired by the design used in the previously published papers [3, 4]. First, we generated the finite population FP of size N=500 000 that contains:

1. The vector of four auxiliary variables $x={\left(x_{1},x_{2},x_{3},x_{4}\right)}^{⊤}$ generated as
$$\left\{\begin{array}{l} x_{1i}=\nu_{1i}\\ x_{2i}=\nu_{2i}+0.3x_{1i}\\ x_{3i}=\nu_{3i}+0.2(x_{1i}+x_{2i})\\ x_{4i}=\nu_{4i}+0.1(x_{1i}+x_{2i}+x_{3i}) \end{array}\right.$$
  where $\nu_{1i}∼\mathrm{Bernoulli}(0.5),\;\;\nu_{2i}∼\mathrm{Unif}(0,2),\;\;\nu_{3i}∼\mathrm{Exp}(1),\nu_{4i}∼\chi^{2}(4)$ and
2. The outcome variables: a continuous outcome $y_{i}∼\mathrm{Norm}(\mu_{i};1)$ and a binary outcome $y_{bi}∼\mathrm{Bernoulli}(\mathrm{expit}(\mu_{i}))$, where $\mu_{i}=-x_{1i}-x_{2i}+x_{3i}+x_{4i}$. By this design, $x_{4}$ has the strongest correlation and $x_{1}$ has the weakest correlation with the outcome y (i.e., $\rho (y,x_{4})\approx 0.87,\;\;\rho (y,x_{1})\approx -0.14$). Also, the correlation between $y_{b}$ and $x_{4}$ is about three times smaller than the correlation between y and $x_{4}$ (i.e., $\rho (y_{b},x_{4})\approx 0.30$). The two parameters of interest are: $\mu =\sum_{i\in \text{FP}}y_{i}/N$ for the continuous outcome (population mean) and $\mu =\sum_{i\in \text{FP}}y_{bi}/N$ for the binary outcome (population prevalence). In this example, the true values of the target parameters in the finite population were μ=3.975 and μ=0.890 for the continuous and binary outcomes, respectively.

Next, the two probability reference surveys $s_{p_{1}}$ of size $n_{p_{1}}=12\;500$ and $s_{p_{2}}$ of size $n_{p_{2}}=25\;000$ were sampled independently from FP. The sample $s_{p_{1}}$ was selected using Poisson sampling with inclusion probabilities $n_{p_{1}}q_{i}/\sum_{i\in \text{FP}}q_{i}$, where $q_{i}={\mathrm{const}}_{1}+x_{3i}+0.03y_{i}$ for i∈FP. The sample $s_{p_{2}}$ was selected using the randomized systematic probability proportional to size (PPS) sampling method [17, 18] with inclusion probabilities proportional to $z_{i}={\mathrm{const}}_{2}+x_{2i}$, i∈FP. We set ${\mathrm{const}}_{1}=0.97$ and ${\mathrm{const}}_{2}=0.05$ to ensure sufficient variation in the sampling weights.

Finally, the nonprobability sample $s_{c}$ of size $n_{c}$ was sampled from FP, independently of $s_{p_{1}}$ and $s_{p_{2}}$, using Poisson sampling with the participation probabilities defined by the model $\pi^{c}(x_{i},\beta )=\exp(\beta^{⊤}x_{i})$ with $\beta ={\left(\beta_{0},{\tilde{\beta }}^{⊤}\right)}^{⊤}$ and $\tilde{\beta }=(0.18,0.18,-0.27,-0.27)$. The choice of $\beta_{0}$ is determined by the desired sample size. For this study, we set $\beta_{0}=-4.33$, which corresponds to the expected sample size $n_{c}=2500$ that is similar to the size of the nonprobability sample in the real data application in Section 7. The choice of β coefficients ensures that $0<\pi^{c}(x_{i},\beta )<1$ for each i, and implies $\rho (\pi^{c},x_{4})\approx -0.81$ and $\rho (\pi^{c},x_{1})\approx 0.10$.

By design, the outcome variable is available in the nonprobability sample $s_{c}$ but not in the reference surveys $s_{p_{1}}$ and $s_{p_{2}}$. The four auxiliary variables are available in the nonprobability sample $s_{c}$. The availability of the auxiliary variables in each of the two reference surveys is defined by different scenarios as described in Section 6.1. The two reference surveys also contain the sampling weights equal to the inverse of their respective inclusion probabilities.

### 6.1 Simulation Scenarios

We focused our attention on the following four scenarios:

1. Case 0: $s_{p_{1}}$ and $s_{p_{2}}$ surveys contain $\left\{x_{1},x_{2},x_{3},x_{4}\right\}$; that is, $x^{\left(p_{2}\right)}=(1,x_{1},x_{2},x_{3},x_{4})$.
2. Case PC.1: $s_{p_{2}}$ survey contains $\left\{x_{3},x_{4}\right\}$ and $s_{p_{1}}$ survey contains $\left\{x_{1},x_{2}\right\}$; that is, $x^{\left(p_{2}\right)}=(1,x_{3},x_{4})$ and $x^{\left(p_{1}\right)}=(x_{1},x_{2})$.
3. Case PC.2: $s_{p_{2}}$ survey contains $\left\{x_{2},x_{3},x_{4}\right\}$ and $s_{p_{1}}$ survey contains $\left\{x_{1},x_{2},x_{3}\right\}$; that is, $x^{\left(p_{2}\right)}=(1,x_{2},x_{3},x_{4})$ and $x^{\left(p_{1}\right)}=x_{1}$.
4. Case PC.3: $s_{p_{2}}$ survey contains $\left\{x_{1},x_{2},x_{3}\right\}$ and $s_{p_{1}}$ survey contains $\left\{x_{2},x_{3},x_{4}\right\}$; that is, $x^{\left(p_{2}\right)}=(1,x_{1},x_{2},x_{3})$ and $x^{\left(p_{1}\right)}=x_{4}$.

These scenarios were developed with a few goals in mind: (1) to evaluate and compare the performance of various methods using one and two reference surveys under correctly specified models (Case 0); (2) to study the effect of the precalibration procedure described in Section 4.1 on the performance of the raking ratio calibration estimator with two reference surveys; in particular, the performance of the jackknife variance estimator compared to the variance estimator obtained using the analytic formula (12) (Cases PC.1‐PC.3); (3) to study the effect of the omitted covariate in the participation model on the performance of the various estimators (Cases PC.1‐PC.3).

To achieve these goals, we considered the following 8 different estimators for the target parameter (μ):

E1 is the *unweighted* estimator that computes the sample mean using the nonprobability sample $s_{c}$ only; E2 is the *true weights (TW)* estimator that utilizes the true sampling weights used to generate the nonprobability sample $s_{c}$; E3 is the *ALP* $s_{p_{1}}$ estimator that utilizes the pseudo‐weights estimated using the ALP approach with the reference survey $s_{p_{1}}$; E4 is the *CLW* $s_{p_{1}}$ estimator that utilizes the pseudo‐weights estimated using the CLW approach with the reference survey $s_{p_{1}}$; E5 is the *calibration* $s_{p_{1}}$ estimator that utilizes the pseudo‐weights estimated using the raking ratio calibration method (Section 3.1.2) with the reference survey $s_{p_{1}}$; E6 is the *calibration* $s_{p_{2}}$ estimator that utilizes the pseudo‐weights estimated using the raking ratio calibration method (Section 3.1.2) with the reference survey $s_{p_{2}}$; E7 is the *calibration* $s_{p_{2}},s_{p_{1}}$ estimator that utilizes the pseudo‐weights estimated using the raking ratio calibration approach (Section 4) with two reference surveys $s_{p_{1}}$ and $s_{p_{2}}$; E8 is the *calibration‐PC* $s_{p_{2}},s_{p_{1}}$ estimator that utilizes the pseudo‐weights estimated using the raking ratio calibration approach (Section 4) with two reference surveys $s_{p_{1}}$ and $s_{p_{2}}$ and the precalibration procedure applied to match the population totals (including the sum of the weights) of the overlapping auxiliary variables in $s_{p_{1}}$ to those of $s_{p_{2}}$.

Estimators E1 and E2 were computed only for Case 0 because they do not use data from the reference surveys. The remaining estimators E3‐E8 were computed for each scenario. In Case 0, estimators E7‐E8 are essentially the same estimators as E6 because all four auxiliary variables were observed in $s_{p_{2}}$, the largest of the two reference surveys. In Case PC.1, the two reference surveys do not contain overlapping covariates, in which case the precalibration procedure was used to ensure that the sum of the weights in $s_{p_{1}}$ is equal to the sum of the weights in $s_{p_{2}}$.

### 6.2 Evaluation Metrics

Each simulation experiment was repeated B=4000 times for each scenario to obtain the b‐th estimated value (b=1,…,B) of the population mean ${\hat{\mu }}^{\left(b\right)}$, the variance estimator ${\hat{v}}^{\left(b\right)}$ using the analytic formula, and the delete‐a‐group jackknife variance estimator ${\hat{v}}_{\mathrm{JK}}^{\left(b\right)}$ for each estimation method. For the delete‐a‐group jackknife estimator, we used 50 random groups in each stratum to optimize the computational time.

To evaluate the performance of the point estimators, the relative bias (%RB) and the mean squared error (MSE) were computed as follows:

$$\%\mathrm{RB}=\frac{1}{B}\overset{B}{\underset{b=1}{\sum }}\frac{{\hat{\mu }}^{\left(b\right)}-\mu }{\mu }\times 100,\;\;\mathrm{MSE}=\frac{1}{B}\overset{B}{\underset{b=1}{\sum }}{\left({\hat{\mu }}^{\left(b\right)}-\mu \right)}^{2}$$

To evaluate the performance of the variance estimators that rely on the analytic formulae, we computed the standard error ratio SER and the coverage probability %CP as follows:

$$\mathrm{SER}=\frac{\sqrt{\frac{1}{B}\sum_{b=1}^{B}{\hat{v}}^{\left(b\right)}}}{\sqrt{V}},\;\;\mathrm{CP}=\frac{1}{B}\overset{B}{\underset{b=1}{\sum }}𝕀(\mu \in {\text{CI}}^{\left(b\right)})\times 100$$

where $V=\frac{1}{B-1}\sum_{b=1}^{B}{\left[{\hat{\mu }}^{\left(b\right)}-\frac{1}{B}\sum_{b=1}^{B}{\hat{\mu }}^{\left(b\right)}\right]}^{2}$ is the empirical (‘true’) variance of $\hat{\mu }$ and ${\text{CI}}^{\left(b\right)}=({\hat{\mu }}^{\left(b\right)}-z_{1-\alpha /2}\sqrt{{\hat{v}}^{\left(b\right)}},{\hat{\mu }}^{\left(b\right)}+z_{1-\alpha /2}\sqrt{{\hat{v}}^{\left(b\right)}})$ for α=0.05. To evaluate the performance of the delete‐a‐group jackknife variance estimator and compare its performance with the variance estimator obtained using the analytic formulae, we computed ${\mathrm{SER}}_{\mathrm{JK}}$ and ${\mathrm{CP}}_{\mathrm{JK}}$ that rely on the variance estimator ${\hat{v}}_{\mathrm{JK}}^{\left(b\right)}$.

### 6.3 Simulation Results

The results for all four scenarios are presented in Tables 1, 2, 3, 4. Each table contains the results for the continuous outcome followed by the results for the binary outcome.

**TABLE 1:** Case 0: $s_{p_{1}}$ and $s_{p_{2}}$ surveys contain four auxiliary variables $\left\{x_{1},x_{2},x_{3},x_{4}\right\}$. Mean relative bias (RB, %), variance (V – using analytic formula, $V_{\mathrm{JK}}$—using jackknife estimator), ratio of the estimated standard error to the simulated standard error (SER—using analytic variance formula, ${\mathrm{SER}}_{\mathrm{JK}}$—using jackknife variance estimator), mean squared error (MSE), and coverage probability (CP—using analytic variance formula, ${\mathrm{CP}}_{\mathrm{JK}}$—using jackknife variance estimator) for various estimators of the population mean μ obtained from 4000 simulated non‐probability samples $s_{c}$ of size $n_{c}=2500$ ($f_{c}=0.5\%$).

Estimator	Continuous outcome							
	RB	$V\times 10^{2}$	$V_{\mathrm{JK}}\times 10^{2}$	SER	${\mathrm{SER}}_{\mathrm{JK}}$	$\mathrm{MSE}\times 10^{2}$	CP	${\mathrm{CP}}_{\mathrm{JK}}$
Unweighted	−42.78	0.22	0.22	1.02	1.02	289.36	0.00	0.00
TW	−0.19	4.43	4.85	0.78	0.79	4.83	90.30	90.40
ALP $s_{p_{1}}$	−0.12	3.91	4.62	0.74	0.76	4.34	89.00	89.20
CLW $s_{p_{1}}$	0.00	4.02	4.76	0.73	0.75	4.47	89.47	89.60
Calibration $s_{p_{1}}$	0.01	0.15	0.16	1.00	1.02	0.15	95.10	95.40
Calibration $s_{p_{2}}$	0.00	0.14	0.15	0.97	0.99	0.15	94.07	94.03
Calibration $s_{p_{2}},s_{p_{1}}$	0.00	0.14	0.15	0.96	0.99	0.15	93.87	94.03
Calibration‐PC $s_{p_{2}},s_{p_{1}}$	0.00	0.14	0.15	0.96	0.99	0.15	93.87	94.03

	Binary outcome							
Estimator	RB	$V\times 10^{5}$	$V_{\mathrm{JK}}\times 10^{5}$	SER	${\mathrm{SER}}_{\mathrm{JK}}$	$\mathrm{MSE}\times 10^{5}$	CP	${\mathrm{CP}}_{\mathrm{JK}}$
Unweighted	−10.14	6.40	6.41	1.00	1.00	820.46	0.00	0.00
TW	−0.01	3.08	3.11	0.99	0.99	3.12	94.85	94.23
ALP $s_{p_{1}}$	0.00	2.34	2.38	0.99	1.00	2.37	94.57	94.35
CLW $s_{p_{1}}$	0.00	2.35	2.39	0.99	1.00	2.37	94.55	94.30
Calibration $s_{p_{1}}$	0.12	3.46	4.06	0.93	0.99	4.11	91.57	92.48
Calibration $s_{p_{2}}$	0.12	3.45	4.05	0.93	0.98	4.09	91.63	92.48
Calibration $s_{p_{2}},s_{p_{1}}$	0.12	3.45	4.05	0.93	0.98	4.09	91.58	92.48
Calibration‐PC $s_{p_{2}},s_{p_{1}}$	0.12	3.45	4.05	0.93	0.98	4.09	91.58	92.48

*Note:* TW–true weights, PC–precalibration.

**TABLE 2:** Case PC.1: $s_{p_{1}}$ survey contains auxiliary variables $\left\{x_{1},x_{2}\right\}$; $s_{p_{2}}$ survey contains auxiliary variables $\left\{x_{3},x_{4}\right\}$. Mean relative bias (RB, %), variance (V – using analytic formula, $V_{\mathrm{JK}}$ – using jackknife estimator), ratio of the estimated standard error to the simulated standard error (SER – using analytic variance formula, ${\mathrm{SER}}_{\mathrm{JK}}$ – using jackknife variance estimator), mean squared error (MSE), and coverage probability (CP – using analytic variance formula, ${\mathrm{CP}}_{\mathrm{JK}}$ – using jackknife variance estimator) for various estimators of the population mean μ obtained from 4000 simulated non‐probability samples $s_{c}$ of size $n_{c}=2500$ ($f_{c}=0.5\%$).

Estimator	Continuous outcome							
	RB	$V\times 10^{2}$	$V_{\mathrm{JK}}\times 10^{2}$	SER	${\mathrm{SER}}_{\mathrm{JK}}$	$\mathrm{MSE}\times 10^{2}$	CP	${\mathrm{CP}}_{\mathrm{JK}}$
ALP $s_{p_{1}}$	−41.52	0.21	0.21	1.00	0.99	272.65	0.00	0.00
CLW $s_{p_{1}}$	−41.52	0.21	0.21	1.00	0.99	272.65	0.00	0.00
Calibration $s_{p_{1}}$	−41.53	0.21	0.21	1.00	0.99	272.68	0.00	0.00
Calibration $s_{p_{2}}$	−3.32	0.19	0.20	0.97	0.99	1.94	15.17	16.07
Calibration $s_{p_{2}},s_{p_{1}}$	0.02	0.17	0.19	0.97	1.02	0.18	94.53	95.53
Calibration‐PC $s_{p_{2}},s_{p_{1}}$	0.02	0.17	0.15	1.07	1.01	0.15	96.67	95.23

	Binary outcome							
Estimator	RB	$V\times 10^{5}$	$V_{\mathrm{JK}}\times 10^{5}$	SER	${\mathrm{SER}}_{\mathrm{JK}}$	$\mathrm{MSE}\times 10^{5}$	CP	${\mathrm{CP}}_{\mathrm{JK}}$
ALP $s_{p_{1}}$	−9.48	5.98	6.05	1.02	1.02	718.67	0.00	0.00
CLW $s_{p_{1}}$	−9.48	5.98	6.05	1.02	1.02	718.67	0.00	0.00
Calibration $s_{p_{1}}$	−9.49	5.98	6.05	1.02	1.02	718.77	0.00	0.00
Calibration $s_{p_{2}}$	−0.85	3.67	4.24	0.93	0.98	9.90	77.00	80.00
Calibration $s_{p_{2}},s_{p_{1}}$	0.12	3.55	4.22	0.93	0.99	4.22	91.90	92.67
Calibration‐PC $s_{p_{2}},s_{p_{1}}$	0.12	3.55	4.07	0.94	0.99	4.08	92.43	92.57

*Note:* PC–precalibration.

**TABLE 3:** Case PC.2: $s_{p_{1}}$ survey contains auxiliary variables $\left\{x_{1},x_{2},x_{3}\right\}$, $s_{p_{2}}$ survey contains auxiliary variables $\left\{x_{2},x_{3},x_{4}\right\}$. Mean relative bias (RB, %), variance (V – using analytic formula, $V_{\mathrm{JK}}$ – using jackknife estimator), ratio of the estimated standard error to the simulated standard error (SER – using analytic variance formula, ${\mathrm{SER}}_{\mathrm{JK}}$ – using jackknife variance estimator), mean squared error (MSE), and coverage probability (CP – using analytic variance formula, ${\mathrm{CP}}_{\mathrm{JK}}$ – using jackknife variance estimator) for various estimators of the population mean μ obtained from 4000 simulated non‐probability samples $s_{c}$ of size $n_{c}=2500$ ($f_{c}=0.5\%$).

Estimator	Continuous outcome							
	RB	$V\times 10^{2}$	$V_{\mathrm{JK}}\times 10^{2}$	SER	${\mathrm{SER}}_{\mathrm{JK}}$	$\mathrm{MSE}\times 10^{2}$	CP	${\mathrm{CP}}_{\mathrm{JK}}$
ALP $s_{p_{1}}$	−35.26	0.24	0.25	1.00	1.00	196.72	0.00	0.00
CLW $s_{p_{1}}$	−35.26	0.24	0.25	1.00	1.00	196.67	0.00	0.00
Calibration $s_{p_{1}}$	−35.25	0.20	0.20	1.01	1.01	196.57	0.00	0.00
Calibration $s_{p_{2}}$	−1.06	0.16	0.16	1.00	1.01	0.33	80.00	81.00
Calibration $s_{p_{2}},s_{p_{1}}$	−0.02	0.15	0.15	1.00	1.02	0.15	95.53	95.90
Calibration‐PC $s_{p_{2}},s_{p_{1}}$	−0.02	0.15	0.15	1.01	1.02	0.14	95.77	95.63

	Binary outcome							
Estimator	RB	$V\times 10^{5}$	$V_{\mathrm{JK}}\times 10^{5}$	SER	${\mathrm{SER}}_{\mathrm{JK}}$	$\mathrm{MSE}\times 10^{5}$	CP	${\mathrm{CP}}_{\mathrm{JK}}$
ALP $s_{p_{1}}$	−7.30	4.97	4.99	1.02	1.02	427.63	0.00	0.00
CLW $s_{p_{1}}$	−7.30	4.97	4.99	1.02	1.02	427.61	0.00	0.00
Calibration $s_{p_{1}}$	−7.30	5.08	5.12	1.01	1.01	426.96	0.00	0.00
Calibration $s_{p_{2}}$	−0.18	3.51	4.06	0.95	1.00	4.13	93.60	94.33
Calibration $s_{p_{2}},s_{p_{1}}$	0.12	3.45	4.01	0.94	0.99	4.00	92.53	92.80
Calibration‐PC $s_{p_{2}},s_{p_{1}}$	0.12	3.45	3.99	0.94	0.99	3.98	92.50	92.80

*Note:* PC–precalibration.

**TABLE 4:** Case PC.3: $s_{p_{1}}$ survey contains auxiliary variables $\left\{x_{2},x_{3},x_{4}\right\}$ and $s_{p_{2}}$ contains auxiliary variables $\left\{x_{1},x_{2},x_{3}\right\}$. Mean relative bias (RB, %), variance (V – using analytic formula, $V_{\mathrm{JK}}$ – using jackknife estimator), ratio of the estimated standard error to the simulated standard error (SER – using analytic variance formula, ${\mathrm{SER}}_{\mathrm{JK}}$ – using jackknife variance estimator), mean squared error (MSE), and coverage probability (CP – using analytic variance formula, ${\mathrm{CP}}_{\mathrm{JK}}$ – using jackknife variance estimator) for various estimators of the population mean μ obtained from 4000 simulated non‐probability samples $s_{c}$ of size $n_{c}=2500$ ($f_{c}=0.5\%$).

Estimator	Continuous outcome							
	RB	$V\times 10^{2}$	$V_{\mathrm{JK}}\times 10^{2}$	SER	${\mathrm{SER}}_{\mathrm{JK}}$	$\mathrm{MSE}\times 10^{2}$	CP	${\mathrm{CP}}_{\mathrm{JK}}$
ALP $s_{p_{1}}$	−1.48	3.25	3.56	0.81	0.82	3.77	80.53	80.60
CLW $s_{p_{1}}$	−1.37	3.33	3.66	0.80	0.82	3.82	81.47	81.23
Calibration $s_{p_{1}}$	−1.07	0.17	0.18	0.96	0.98	0.36	80.43	81.20
Calibration $s_{p_{2}}$	−35.22	0.20	0.21	1.01	1.01	196.24	0.00	0.00
Calibration $s_{p_{2}},s_{p_{1}}$	0.01	0.33	0.41	0.98	1.10	0.34	94.67	96.60
Calibration‐PC $s_{p_{2}},s_{p_{1}}$	−0.01	0.33	0.16	1.42	0.99	0.16	99.53	94.60

	Binary outcome							
Estimator	RB	$V\times 10^{5}$	$V_{\mathrm{JK}}\times 10^{5}$	SER	${\mathrm{SER}}_{\mathrm{JK}}$	$\mathrm{MSE}\times 10^{5}$	CP	${\mathrm{CP}}_{\mathrm{JK}}$
ALP $s_{p_{1}}$	−0.33	2.49	2.54	0.99	0.99	3.41	91.07	90.77
CLW $s_{p_{1}}$	−0.33	2.50	2.55	0.99	0.99	3.41	91.17	90.80
Calibration $s_{p_{1}}$	−0.19	3.55	4.16	0.93	0.98	4.37	93.47	94.30
Calibration $s_{p_{2}}$	−7.29	5.10	5.15	0.99	0.99	426.79	0.00	0.00
Calibration $s_{p_{2}},s_{p_{1}}$	0.12	3.66	4.37	0.93	0.99	4.32	92.37	93.27
Calibration‐PC $s_{p_{2}},s_{p_{1}}$	0.12	3.66	4.10	0.95	0.99	4.08	93.07	92.37

*Note:* PC–precalibration.

#### 6.3.1 Case 0

In terms of bias, the results for Case 0 (Table 1) show that all the estimators, except the unweighted estimator, have satisfactory performance. The unweighted estimator has a large negative bias, as expected by design. In terms of variance estimation, the estimated standard errors of the raking ratio calibration estimators for the continuous outcome appeared very close to the simulated standard errors (SER and ${\mathrm{SER}}_{\mathrm{JK}}$ are both close to 1), leading to optimal coverage probabilities (95%). This was not the case for the estimator with true weights and the ALP and CLW estimators for the continuous outcome. For these three estimators, we observed underestimated standard errors and less than optimal coverage probabilities. Our investigation showed that these estimators do not perform well in situations where the true sampling weights associated with the nonprobability sample are very dispersed. For more details, see Appendix C. The values of SER and ${\mathrm{SER}}_{\mathrm{JK}}$ were very close to or equal to 1 for all the estimators for the binary outcome, indicating satisfactory performance in the case of very dispersed weights for this type of outcome.

The results in Table 1 indicate that compared to the ALP and CLW estimators, the calibration estimators have markedly smaller variances for the continuous outcome but larger variances for the binary outcome. The profound reduction in variance of the calibration estimators for the continuous outcome is likely explained by the linear relationship between the outcome and the auxiliary variables. Kim and Riddles [19] proved the optimality of the classic calibration estimator that relies on (known) population totals in the framework of the superpopulation linear regression model.

#### 6.3.2 Cases PC.1–PC.3

The results for the cases PC.1–PC.3 (Tables 2, 3, 4) show that the precalibration procedure does not have an effect on the bias of the mean estimator for both continuous and binary outcomes. In terms of variance estimation, the jackknife variance estimator performs reasonably well in all three cases, demonstrating that it successfully accounts for the potential correlation between the reference surveys introduced by the precalibration procedure, which is not accounted for by the analytic formula (12). In addition, the precalibration procedure tends to somewhat improve the efficiency of the estimators without an increase in bias. Both observations are demonstrated in Case PC.3 (Table 4).

The simulation results in Tables 2, 3, 4 also provide insight into the effect of the omitted variable on the bias of various estimators. We observed satisfactory performance of the calibration estimator with two reference surveys in all scenarios (bias in the range of −0.02 to 0.12%), which was expected since the four auxiliary variables were always available from the two reference surveys combined. Estimators that utilize only one reference survey demonstrate a sizable bias which is significantly larger for scenarios in which the variable $x_{4}$ is omitted as opposed to scenarios in which the variable $x_{1}$ is omitted from the reference survey: for example, the bias is approximately −35% in PC.2 (Table 3) as opposed to up to −1.5% in PC.3 (Table 4) for the estimators that rely on $s_{p_{1}}$. Importantly, in the case of the calibration estimators, the size of the bias was not affected by the size of the reference survey: the bias for the *calibration* $s_{p_{2}}$ estimator in PC.2 (Table 3) is practically identical to the bias of the *calibration* $s_{p_{1}}$ estimator in PC.3 (Table 4) and vice versa, despite the fact that $n_{p_{2}}$ is twice as large as $n_{p_{1}}$ by design. The observed pattern is very similar for both continuous and binary outcomes.

One additional simulation scenario is presented in Appendix D. It corresponds to the Case PC.3 but with participation model defined by $\pi^{c}(x_{i},\beta )=\exp(\beta^{⊤}x_{i})$ with $\beta ={\left(\beta_{0},{\tilde{\beta }}^{⊤}\right)}^{⊤}$, $\tilde{\beta }=(0.18,0.18,0,0)$ and $\beta_{0}=-5.61$ (to obtain $n_{c}=2500$). In this case, the participation probabilities are only weakly correlated with the outcome y, leading to a very small bias in the unweighted estimator. Nevertheless, the results demonstrate that the variances of the pseudo‐weighted mean estimates were smaller than the variance of the unweighted estimator for both continuous and binary outcome and for all the estimators (E3‐E8) discussed in this paper. These results confirm the importance of using the pseudo‐weighted estimators even in situations when the unweighted estimators are believed to be unbiased.

## 7 Real Data Example

In this section, we demonstrated the application of the methods described above to real‐world data collected via a national Canadian survey of approximately 2000 workers (hereafter, ‘cannabis sample’). The original purpose of this survey was to collect information on workers' cannabis use and perceptions of cannabis use at work before the legalization of non‐medical cannabis use in Canada in October 2018. A detailed description of the sample is available from Carnide et al. [20]. The target population of the study consisted of Canadians at least 18 years of age, currently employed, and working at least 15 or more hours per week in workplaces with at least 5 employees. Due to time and funding constraints, eligible volunteers were recruited in June 2018 from an existing household panel of over 100,000 Canadians maintained by a private research firm.

We performed preliminary descriptive analyses to compare (unweighted) prevalence estimates for selected auxiliary variables from the nonprobability cannabis sample and the corresponding survey‐weighted prevalence estimates from two large‐scale population‐based reference surveys: (1) the Canadian Tobacco, Alcohol and Drugs Survey (CTADS, n=7390) [21] and (2) the Labour Force Survey (LFS, n=50 294) [22], collected around the same time as the cannabis sample. The estimates presented in Appendix E demonstrate sizable discrepancies in the distributions of participants' age, cannabis use in the past 12 months, perceived health, education level, and workplace size. These findings suggest that the decision to participate in the cannabis sample can be influenced by various demographic, socioeconomic, health, and lifestyle characteristics, as well as characteristics related to work. These characteristics must be taken into account using the methods described in this paper to improve the generalizability of the inference from the cannabis sample to the target population. Methods that can accommodate multiple reference surveys are particularly appealing in this application, since none of the existing reference surveys contain all the important auxiliary variables that may influence the individual's decision to participate (e.g., no single available reference survey contains the information on both health/lifestyle and workplace characteristics).

To demonstrate the application of these methods, we aimed to estimate the prevalence of work‐related injury (self‐reported), overall and stratified by safety‐sensitive and non‐safety‐sensitive jobs, from the cannabis sample of individuals who provided complete data on all the variables used in this analysis (n=1993 participants in total, approximately half of whom reported working in safety‐sensitive jobs). Establishing this baseline (prelegalization) prevalence is vital for future studies of the policy effects on people's health and safety.

The application of the methods described in this paper starts from fitting a participation model to estimate the unknown participation probabilities and, subsequently, the pseudo‐weights. The auxiliary variables were included in the participation model if they (1) were plausible determinants of the individual's decision to participate in the cannabis survey; (2) were potentially associated with the outcome(s) of interest; and (3) were available in both the cannabis sample and at least one of the reference surveys. In our study, the variables age, sex, cannabis use in the past 12 months, perceived health, education, hours of work per week, job permanency, and establishment size met these requirements and were included in modeling and estimating the participation rates.

Next, we estimated the prevalence of work‐related injury (overall and stratified by safety‐sensitive jobs) and their corresponding standard error using the ALP, CLW and raking ratio calibration methods with one reference survey (3 estimates using the CTADS survey and 3 estimates using the LFS survey), as well as using the raking ratio calibration method with two reference surveys (2 estimates with and without the precalibration of the overlapping auxiliary variables). The sum of the weights in the CTADS survey was precalibrated to match the sum of the weights in the LFS survey for both estimates that use two reference surveys in agreement with our recommendations in Section 4.1. The overlapping auxiliary variables (sex, age, and education) were used from the LFS and their totals in the CTADS were precalibrated to match the estimated totals in the LFS. The standard errors estimates were obtained using the corresponding analytic formulae developed for each method. The ${𝕍}_{c}$ component of the variance estimator was obtained under the Poisson sampling assumption for the cannabis sample. The ${𝕍}_{p_{k}}$ components of the variance estimator were obtained using bootstrap weights provided by Statistics Canada to reflect the information about multi‐stage complex sampling design used in the CTADS and LFS surveys (500 bootstrap weights for the CTADS survey and 1000 bootstrap weights for the LFS survey) [21, 22].

The results are summarized in Table 5. Overall, we did not observe significant discrepancies of practical importance between the point estimators using various pseudo‐weighted approaches and the unweighted point estimator for this specific outcome. The standard error estimates for the prevalence of work‐related injury in safety‐sensitive jobs were twice as high for all the pseudo‐weighted estimators compared to the unweighted estimator. The standard errors for other pseudo‐weighted prevalence estimates for work‐related injury (overall and non‐safety‐sensitive job) were only slightly larger than the standard errors of the corresponding unweighted estimators. Also, the standard errors of the pseudo‐weighted estimators in Table 5 are practically identical across various estimation methods.

**TABLE 5:** Unweighted and pseudo‐weighted estimates of the prelegalization work‐related injury prevalence (mean, %) and their corresponding standard errors (SE) obtained from the cannabis sample (n=1993) using various methods with one or two reference surveys.

	Outcome: Work‐related injury					
Estimator	Overall		Safety‐sensitive work		Non‐safety‐sensitive work	
	mean	SE	mean	SE	mean	SE
Unweighted	7.78	0.06	13.93	0.08	1.70	0.03
ALP 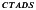	7.96	0.08	14.45	0.15	1.38	0.04
CLW 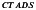	7.96	0.08	14.45	0.15	1.38	0.04
Calibration 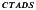	7.77	0.08	14.15	0.15	1.38	0.04
ALP 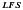	8.69	0.09	14.98	0.15	1.73	0.05
CLW 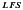	8.69	0.09	14.98	0.15	1.73	0.05
Calibration 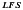	8.61	0.08	14.84	0.14	1.71	0.04
Calibration 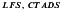	7.76	0.08	14.09	0.16	1.36	0.04
Calibration‐PC 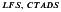	7.85	0.08	14.01	0.15	1.47	0.04

*Note:* PC–pre‐calibration.

Although the point estimates generated by the different methods are similar to one another and to the unweighted estimate for this particular outcome, estimates may look different for other outcomes. It may not be possible to determine a priori whether the discrepancies between the pseudo‐weighted and unweighted estimates will be practically important. In addition, underestimated standard errors will result in too narrow confidence intervals and inflated type I errors. As such, applying the methods proposed in this paper to account for participation bias is warranted when working with data from nonprobability samples.

## 8 Discussion

In this paper, we presented a general statistical framework for bias correction in estimating mean (prevalence) from nonprobability samples. This framework enables us to estimate the unknown participation probabilities in the nonprobability sample and construct pseudo‐weighted Hajek‐type estimators for mean (prevalence) in the finite population. To estimate the unknown participation probabilities, the system of estimating equations is assembled by equating the finite population totals estimated using data from the nonprobability sample and unknown participation weights, with their counterparts, estimated using data from the existing probability survey(s) and the provided sampling weights. The resulting estimating equations are solved with respect to the parameters in the assumed participation model, which are further used to estimate the so‐called pseudo‐weights. The previously published ALP and CLW methods that rely on one reference survey are special cases within this framework. We devoted special attention to the calibration estimation approach, another important special case within the proposed framework. This method can be viewed as a natural extension of the classic calibration method, popular in survey sampling, in which the pseudo‐weights play the role of the sampling weights to be calibrated and known population totals are replaced with the estimates from available probability survey(s). The described framework is flexible in the sense that it can integrate multiple reference surveys into the estimating equations. This flexibility is very important in real‐world applications where the variables that determine the individual participation in the nonprobability sample cannot be found in a single reference survey. This was our experience when analyzing the data from the cannabis sample that motivated this study.

The consistency and asymptotic properties of the resulting estimators follow from the unbiasedness of the estimating equations under the models that define the participation in the nonprobability sample and in the reference survey(s). In addition, we used the method of Taylor deviates to derive the analytic formulae for the variance estimators of the pseudo‐weighted means and described a leave‐one‐out jackknife estimator, a replication method. Both methods properly account for all sources of variability in the derivation of pseudo‐weights. The jackknife estimator also accounts for potential correlation between the reference surveys introduced by the precalibration procedure aimed at addressing the overlapping auxiliary variables in multiple reference surveys.

We evaluated the performance of various estimators in the simulation study. Our simulation results demonstrated that, in contrast to the ALP and CLW estimators, a calibration estimator is very efficient for continuous outcomes and has very good performance for both continuous and binary outcomes in situations with highly dispersed pseudo‐weights in the nonprobability sample. Additionally, assembling the estimating equations for the calibration estimator does not require access to the reference survey microdata for auxiliary variables, for which the estimated totals and their standard errors are disseminated to the general public (e.g., on a government agency website). This flexibility can be helpful in expanding the set of auxiliary variables to be included in the participation model.

We demonstrated the application of the methods in a real‐world context to establish a baseline prevalence of work‐related injury in the Canadian workforce before cannabis legalization. It appears that for this outcome variable, the pseudo‐weighted point estimates obtained by various methods did not reveal any discrepancies of practical importance in comparison with the unweighted estimate. Although reassuring for this particular outcome, this finding does not diminish the importance of applying the proposed methods to nonprobability samples. In a multi‐outcome study, like the Cannabis study, it is not unlikely to discover larger discrepancies between the estimates when applying the same set of the estimated pseudo‐weights to estimating mean (prevalence) of other outcomes of interest. On the other hand, one cannot exclude the possibility of omitting an important variable that is strongly associated with both survey participation and outcomes of interest. If this is the case, the resulting point estimates using pseudo‐weights might not fully correct for participation bias. For example, in previously published analyses, we found that workers in safety‐sensitive jobs were more likely to report workplace cannabis use [20] and that workplace cannabis use (but not non‐workplace‐use) was associated with workplace injury risk [23]. Although we accounted for cannabis use overall, we could not account for cannabis use at work or safety‐sensitive work in modeling the participation probabilities because they were not measured in any of the available reference surveys. In addition, the standard errors of the pseudo‐weighted estimates for the prevalence of work‐related injury in safety‐sensitive jobs were twice as high for all the pseudo‐weighted estimators compared to the unweighted estimator. The standard errors of the estimates for other categories (overall and non‐safety‐sensitive jobs) were only slightly larger for the pseudo‐weighted estimates compared to the standard errors of the corresponding unweighted estimators. These findings demonstrate the importance of applying the proposed methods for point and variance estimation not only to minimize participation bias but also to properly account for all sources of variability in the point estimates.

## Funding

This work was supported by the Canadian Institutes of Health Research (Grant No. PJT‐186242).

## Conflicts of Interest

The authors declare no conflicts of interest.

## Data Availability

The data that support the findings of this study cannot be shared due to privacy and ethical restrictions. The R code used for the simulation study is available by request from the corresponding author.

## Appendix A Derivation of the Finite Population Variance of $\hat{\mu }$

To simplify the notation, define: $\pi_{i}^{c}(\beta ):=\pi^{c}(x_{i},\beta )$, $w_{i}(\beta ):=w(x_{i},\beta )$ and $h_{i}(\beta ):=h(x_{i},\beta )$. Let $\eta ={\left(\mu ,\beta^{⊤}\right)}^{⊤}$ be the parameter of interest. The estimating equations in (4) can be presented as

$$\Phi (\eta )=\left[\begin{array}{c} U(\mu ,\beta )\\ S(\beta ) \end{array}\right]=0$$

where $U(\mu ,\beta )=N^{-1}\sum_{i=1}^{N}\delta_{i}^{c}w_{i}(\beta )(y_{i}-\mu )$ and $S(\beta )=N^{-1}\sum_{i=1}^{N}\delta_{i}^{c}w_{i}(\beta )h_{i}(\beta )-N^{-1}\sum_{i=1}^{N}\delta_{i}^{p}d_{i}h_{i}(\beta )$.

The finite population variance formula is given by

(A1)
$${𝕍}_{cp}(\hat{\eta })={\left[{𝔼}_{cp}(\phi (\eta_{0}))\right]}^{-1}{𝕍}_{cp}(\Phi (\eta_{0})){\left[{𝔼}_{cp}{\left(\phi (\eta_{0})\right)}^{⊤}\right]}^{-1}$$

where $\phi (\eta )=\frac{\partial \Phi (\eta )}{\partial \eta }$ and $\eta_{0}$ is the true value of η.

We start from computing the ‘bread’ in (A1) and denote ${𝔼}_{cp}(\phi )=\left[\begin{array}{ll} U_{\mu } & U_{\beta }^{⊤}\\ S_{\mu } & S_{\beta } \end{array}\right]$where

$$\begin{array}{ll} & U_{\mu }:={𝔼}_{cp}\left(\frac{\partial U(\mu ,\beta )}{\partial \mu }\right),U_{\beta }:={𝔼}_{cp}\left(\frac{\partial U(\mu ,\beta )}{\partial \beta }\right),\\ & S_{\mu }:={𝔼}_{cp}\left(\frac{\partial S(\beta )}{\partial \mu }\right),S_{\beta }:={𝔼}_{cp}\left(\frac{\partial S(\beta )}{\partial \beta }\right). \end{array}$$

Using the rules for inverse of the 2 × 2 partitioned matrix and the fact that $S_{\mu }:={𝔼}_{cp}(\frac{\partial S}{\partial \mu })=0$ and $U_{\mu }=-1$, we obtain:

$${\left[{𝔼}_{cp}(\phi )\right]}^{-1}=\left[\begin{array}{cc} -\;1 & b^{⊤}\\ 0 & S_{\beta }^{-1} \end{array}\right]$$

where $U_{\beta }=N^{-1}\sum_{i=1}^{N}\pi_{i}^{c}(\beta )(y_{i}-\mu ){\left(\frac{\partial w_{i}(\beta )}{\partial \beta }\right)}^{⊤};S_{\beta }=N^{-1}\sum_{i=1}^{N}\pi_{i}^{c}(\beta )h_{i}(\beta ){\left(\frac{\partial w_{i}(\beta )}{\partial \beta }\right)}^{⊤};\;\text{and}\;b^{⊤}=U_{\beta }S_{\beta }^{-1}.$

Using Assumption 5, the “meat” expression in (A1) can be decomposed as ${𝕍}_{cp}(\Phi )={𝕍}_{c}(\Phi_{1})+{𝕍}_{p}(\Phi_{2})$, where

$\Phi_{1}=\left[\begin{array}{l} N^{-1}\sum_{i=1}^{N}\delta_{i}^{c}w_{i}(\beta )(y_{i}-\mu )\\ N^{-1}\sum_{i=1}^{N}\delta_{i}^{c}w_{i}(\beta )h_{i}(\beta ) \end{array}\right]$ and $\Phi_{2}=\left[\begin{array}{l} 0\\ N^{-1}\sum_{i=1}^{N}\delta_{i}^{p}d_{i}h_{i}(\beta ) \end{array}\right]$.

Under Assumption 4 and using ${𝕍}_{c}\left(\delta_{i}^{c}\right)=\pi_{i}^{c}(\beta )(1-\pi_{i}^{c}(\beta ))$, we get ${𝕍}_{c}(\Phi_{1})=N^{-2}\sum_{i=1}^{N}(w_{i}(\beta )-1)\left[\begin{array}{ll} {\left(y_{i}-\mu \right)}^{2} & \left(y_{i}-\mu )h_{i}(\beta \right)\\ \left(y_{i}-\mu )h_{i}(\beta \right) & h_{i}(\beta )h_{i}{\left(\beta \right)}^{⊤} \end{array}\right]$.

Also, ${𝕍}_{p}(\Phi_{2})=N^{-2}\left[\begin{array}{ll} 0 & 0^{⊤}\\ 0 & D \end{array}\right]$, where $D={𝕍}_{p}\left(\sum_{i=1}^{N}\delta_{i}^{p}d_{i}h_{i}(\beta )\right)$ is determined by the sampling design of the reference survey $s_{p}$.

Finally, putting the ‘bread’ and the ‘meat’ components together in (A1), we obtain the explicit analytic formula for the finite population variance of the Hajek estimator as:

(A2)
$$\begin{array}{ll} {𝕍}_{cp}\left(\hat{\mu }(\hat{\beta })\right) & =N^{-2}\left[\begin{array}{cc} -\;1 & b^{⊤} \end{array}\right]\left({𝕍}_{c}(\Phi_{1})+{𝕍}_{p}(\Phi_{2})\right)\left[\begin{array}{c} -\;1\\ b \end{array}\right]\\ & =N^{-2}\overset{N}{\underset{i=1}{\sum }}(w_{i}(\beta )-1){\left\{y_{i}-\mu -b^{⊤}h_{i}(\beta )\right\}}^{2}+N^{-2}b^{⊤}Db \end{array}$$

## Appendix B Derivation of Taylor Deviates for the Raking Ratio Calibration Estimator of the Mean

To illustrate the method, we derive the variance estimator for the calibration estimator with the weight function ${\hat{w}}_{i}:=\exp(-{\hat{\beta }}^{⊤}x_{i})$ (that corresponds to the raking ratio calibration estimator) with one reference survey.

Using the fact that $\frac{\partial \exp(-{\hat{\beta }}^{⊤}x)}{\partial \hat{\beta }}=-[\exp(-{\hat{\beta }}^{⊤}x)]x=-\hat{w}x$, we can write the expression for $\Delta_{i}(\hat{\mu })$ in (11) as:

(B1)
$$\Delta_{i}(\hat{\mu })=\frac{1}{T_{2}}[{\hat{w}}_{i}z_{i}(y_{i}-\hat{\mu })\delta_{i}^{c}-{\hat{U}}_{\beta }\Delta_{i}(\hat{\beta })]$$

where ${\hat{U}}_{\beta }=\sum_{j\in s_{c}}{\hat{w}}_{j}z_{j}(y_{j}-\hat{\mu })x_{j}^{⊤}$ and $\Delta_{i}(\hat{\beta })$ is derived from the estimating equations below:

$$\begin{array}{ll} 0 & =\Delta_{i}\left[\underset{j\in s_{c}\cup s_{p}}{\sum }{\hat{w}}_{j}x_{j}\delta_{j}^{c}-d_{j}x_{j}\delta_{j}^{p}\right]\\ & ={\hat{w}}_{i}x_{i}\delta_{i}^{c}+\frac{\partial \left[\sum_{j\in s_{c}}{\hat{w}}_{j}x_{j}\right]}{\partial \hat{\beta }}\Delta_{i}(\hat{\beta })-d_{i}x_{i}\delta_{i}^{p}={\hat{w}}_{i}x_{i}\delta_{i}^{c}\\ & \;-\left[\underset{j\in s_{c}}{\sum }{\hat{w}}_{j}x_{j}x_{j}^{⊤}\right]\Delta_{i}(\hat{\beta })-d_{i}x_{i}\delta_{i}^{p} \end{array}$$

Solving the last expression with respect to $\Delta_{i}(\beta )$ results in

$$\Delta_{i}(\hat{\beta })={\hat{S}}_{\beta }^{-1}\left({\hat{w}}_{i}x_{i}\delta_{i}^{c}-d_{i}x_{i}\delta_{i}^{p}\right)$$

where ${\hat{S}}_{\beta }=\sum_{j\in s_{c}}{\hat{w}}_{j}x_{j}x_{j}^{⊤}$.

Let ${\hat{b}}^{⊤}={\hat{U}}_{\beta }{\hat{S}}_{\beta }^{-1}$. Then, after substituting the expression for $\Delta_{i}(\hat{\beta })$ in (B1), we obtain:

(B2)
$$\Delta_{i}(\hat{\mu })=\frac{1}{T_{2}}[{\hat{w}}_{i}(y_{i}z_{i}-\hat{\mu }z_{i}-{\hat{b}}^{⊤}x_{i})\delta_{i}^{c}+{\hat{b}}^{⊤}d_{i}x_{i}\delta_{i}^{p}]$$

## Appendix C Impact of Extreme Weights on the Estimators Performance

To explore the effect of extreme weights on the variances of the point estimators in the case of continuous outcome, we generated 4000 nonprobability samples using three different values of the parameter $\beta ={\left(\beta_{0},{\tilde{\beta }}^{⊤}\right)}^{⊤}$ in the participation model: ${\tilde{\beta }}_{E}=(0.18,0.18,-0.27,-0.27)$, ${\tilde{\beta }}_{\mathrm{Mod}}=(0.18,0.18,-0.27,-0.15)$, ${\tilde{\beta }}_{\mathrm{Mild}}=(0.18,0.18,-0.27,-0.10)$ (and $\beta_{0}$ is determined in each case to obtain $n_{c}=2500$). We obtained the corresponding estimators for Case 0 (continuous outcome) using true weights (TW), ALP, CLW and the raking ratio calibration estimator with $s_{p_{1}}$ as a reference survey, as well as the raking ratio calibration estimator with two reference surveys $s_{p_{1}}$ and $s_{p_{2}}$. We also calculated the quantiles of the distribution of the coefficient of variation (CV) of the true sampling weights of 4000 nonprobability samples for each value of β. The results in Table C.1 below demonstrate that performance of the variance estimators for TW, ALP and CLW methods improves with decrease in the CV of the weights (from extreme to moderate to mild CV). Noticeably, the variance estimators of the means obtained using the calibration methods perform well for all three choices of β. The decrease in bias in the unweighted estimator is explained by a reduced correlation between the ‘strong’ variable $x_{4}$ and the participation probability for different choices of β.

Table C.1. Case 0: $s_{p_{1}}$ and $s_{p_{2}}$ surveys contain four auxiliary variables $\left\{x_{1},x_{2},x_{3},x_{4}\right\}$. Mean relative bias (RB, %), mean squared error (MSE), ratio of the estimated standard error to the simulated standard error (SER), and coverage probability (CP) for various estimators of the population mean μ obtained from 4000 simulated nonprobability samples $s_{c}$ of size $n_{c}=2500$ ($f_{c}=0.5\%$). The vector $\beta ={\left(\beta_{0},{\tilde{\beta }}^{⊤}\right)}^{⊤}$ determines the participation probabilities $\pi^{c}(x,\beta )$ used to generate the nonprobability sample $s_{c}$. The entries of the vector  correspond to the (100%,95%,90%,85%) quantiles of the distribution of the coefficient of variation (CV) of the ‘true’ sampling weights over 4000 nonprobability samples.

**Table:**

**Continuous outcome**: “**Extreme**” **CV**				
$\tilde{\beta }={\left(0.18,0.18,-0.27,-0.27\right)}^{⊤}$;				
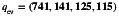
Estimator	RB	SER	$\mathrm{MSE}\times 10^{2}$	CP
Unweighted	−42.77	1.01	289.25	0.00
TW	−0.12	0.80	4.70	90.35
ALP $s_{p_{1}}$	−0.07	0.76	4.16	88.95
CLW $s_{p_{1}}$	0.05	0.75	4.28	89.40
Calibration $s_{p_{1}}$	0.01	0.99	0.16	95.08
Calibration $s_{p_{2}},s_{p_{1}}$	0.01	0.97	0.15	94.08

**Continuous outcome**: “**Moderate**” **CV**				
$\tilde{\beta }={\left(0.18,0.18,-0.27,-0.15\right)}^{⊤}$;				
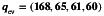
Estimator	RB	SER	$\mathrm{MSE}\times 10^{2}$	CP
Unweighted	−30.69	1.00	149.08	0.00
TW	−0.03	0.96	1.30	94.27
ALP $s_{p_{1}}$	0.00	0.95	0.80	93.95
CLW $s_{p_{1}}$	0.04	0.95	0.81	94.10
Calibration $s_{p_{1}}$	0.01	1.00	0.13	95.08
Calibration $s_{p_{2}},s_{p_{1}}$	0.00	1	0.12	94.90

**Continuous outcome:** ‘**Mild**’ **CV**				
$\tilde{\beta }={\left(0.18,0.18,-0.27,-0.10\right)}^{⊤}$;				
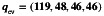
Estimator	RB	SER	$\mathrm{MSE}\times 10^{2}$	CP
Unweighted	−24.23	1.00	93.09	0.00
TW	−0.01	0.98	0.87	94.55
ALP $s_{p_{1}}$	0.01	0.97	0.45	93.95
CLW $s_{p_{1}}$	0.03	0.97	0.46	93.97
Calibration $s_{p_{1}}$	0.01	1.00	0.13	95.1
Calibration $s_{p_{2}},s_{p_{1}}$	0.00	1.00	0.11	95.3

*Note:* TW–true weights.

## Appendix D Simulation Scenario With Negligible Bias

Table D.1. Case PC.3: $s_{p_{1}}$ survey contains auxiliary variables $\left\{x_{2},x_{3},x_{4}\right\}$ and $s_{p_{2}}$ contains auxiliary variables $\left\{x_{1},x_{2},x_{3}\right\}$ and the participation probabilities in $s_{c}$ are defined by the model $\pi^{c}(x_{i},\beta )=\exp(\beta^{⊤}x_{i})$ with $\beta ={\left(\beta_{0},{\tilde{\beta }}^{⊤}\right)}^{⊤}$ and $\tilde{\beta }=(0.18,0.18,0,0)$. Mean relative bias (RB, %), variance using analytic formula (V), ratio of the estimated standard error to the simulated standard error (SER), mean squared error (MSE), and coverage probability (CP) for various estimators of the population mean μ obtained from 4000 simulated nonprobability samples $s_{c}$ of size $n_{c}=2500$ ($f_{c}=0.5\%$).

**Table:**

	Continuous outcome				
Estimator	RB	$V\times 10^{2}$	SER	$\mathrm{MSE}\times 10^{2}$	CP
E1: Unweighted	−2.33	0.42	1.00	1.28	69.23
E3: ALP $s_{p_{1}}$	−0.01	0.42	1.00	0.42	95.03
E4: CLW $s_{p_{1}}$	−0.01	0.42	1.00	0.42	95.03
E5: Calibration $s_{p_{1}}$	0.00	0.42	1.00	0.42	95.00
E6: Calibration $s_{p_{2}}$	−3.31	0.13	0.99	1.86	4.55
E7: Calibration $s_{p_{2}},s_{p_{1}}$	0.00	0.14	1.03	0.13	95.75
E8: Calibration–PC $s_{p_{2}},s_{p_{1}}$	0.00	0.14	1.14	0.11	97.65

	Binary outcome				
Estimator	RB	$V\times 10^{2}$	SER	$\mathrm{MSE}\times 10^{5}$	CP
E1: Unweighted	−0.77	4.12	1.00	8.77	82.12
E3: ALP $s_{p_{1}}$	−0.01	3.72	1.00	3.73	95.23
E4: CLW $s_{p_{1}}$	−0.01	3.72	1.00	3.73	95.23
E5: Calibration $s_{p_{1}}$	−0.01	3.72	1.00	3.73	95.20
E6: Calibration $s_{p_{2}}$	−0.95	3.82	1.00	10.90	73.30
E7: Calibration $s_{p_{2}},s_{p_{1}}$	−0.01	3.51	1.01	3.46	95.15
E8: Calibration–PC $s_{p_{2}},s_{p_{1}}$	0.00	3.51	1.02	3.39	95.38

*Note:* PC–pre‐calibration.

## Appendix E Descriptive Analyses of the Cannabis Sample

Table E.1. Unweighted prevalence estimates (%) estimated from the cannabis sample and survey‐weighted prevalence estimates (%) estimated from the Cannabis, Tobacco, Alcohol and Drugs Survey (CTADS, 2017) and the Labour Force Survey (LFS, June 2018).

**Table:**

Variable	Cannabis sample	CTADS	LFS
	(June 2018)	(Feb–Dec 2017)	(June 2018)
**Demographic**	
Age (in years)			
18–24	6.0	15.1	13.8
25–34	20.8	25.3	24.1
35–44	23.6	22.2	22.2
45–54	25.6	21.6	21.2
55–64	20.2	12.9	15.9
65+	3.8	2.9	2.8
Sex			
Male	55.3	51.5	51.4
Female	44.7	48.5	48.6
**Health/Lifestyle**			
Cannabis use (past 12 months)			
Yes	29.7	18.4	
No	70.3	81.6	
Perceived health			
Excellent	16.2	28.5	
Very good	40.6	41.8	
Good	32.2	26.2	
Fair/poor	11.0	3.5	
**Socioeconomic**			
Education			
Less than post‐secondary	12.0	26.5	25.9
Post‐secondary/ university degree	88.0	73.5	74.1
Hours of work (per week)			
15–30	12.5		15.2
31–40	73.3		71.4
41–50	10.6		9.3
51+	3.6		4.1
Permanent job			
Yes	89.2		86.3
No	10.8		13.7
Workplace size			
< 20 employees	17.3		31.5
20–99 employees	31.7		33.8
100–500 employees	24.7		20.5
> 500 employees	26.3		14.2

## References

[1] J. S. Mindell , S. Giampaoli , A. Goesswald , et al., “Sample Selection, Recruitment and Participation Rates in Health Examination Surveys in Europe–Experience From Seven National Surveys,” BMC Medical Research Methodology 15, no. 1 (2015): 1–19. 26438235 10.1186/s12874-015-0072-4 PMC4595185

[2] F. J. Mölenberg , d C. Vries , A. Burdorf , and v F J. Lenthe , “A Framework for Exploring Non‐Response Patterns Over Time in Health Surveys,” BMC Medical Research Methodology 21, no. 1 (2021): 1–9. 33602123 10.1186/s12874-021-01221-0 PMC7890886

[3] L. Wang , R. Valliant , and Y. Li , “Adjusted Logistic Propensity Weighting Methods for Population Inference Using Nonprobability Volunteer‐Based Epidemiologic Cohorts,” Statistics in Medicine 40, no. 24 (2021): 5237–5250. 34219260 10.1002/sim.9122 PMC8526388

[4] Y. Chen , P. Li , and C. Wu , “Doubly Robust Inference With Nonprobability Survey Samples,” Journal of the American Statistical Association 115, no. 532 (2020): 2011–2021.

[5] D. G. Horvitz and D. J. Thompson , “A Generalization of Sampling Without Replacement From a Finite Universe,” Journal of the American Statistical Association 47, no. 260 (1952): 663–685.

[6] J. Hájek , “Comment on an Essay on the Logical Foundations of Survey Sampling, Part One,” in The Foundations of Survey Sampling, ed. V. P. Godambe and D. A. Sprott (Holt, Rinehart and Winston, 1971), 236.

[7] A. Delevoye and F. Sävje , “Consistency of the Horvitz–Thompson Estimator Under General Sampling and Experimental Designs,” Journal of Statistical Planning and Inference 207 (2020): 190–197.

[8] C. E. Särndal , B. Swensson , and J. Wretman , Model Assisted Survey Sampling (Springer Science & Business Media, 2003).

[9] A. A. Tsiatis , Semiparametric Theory and Missing Data, vol. 4 (Springer, 2006).

[10] D. A. Binder , “On the Variances of Asymptotically Normal Estimators From Complex Surveys,” International Statistical Review 51, no. 3 (1983): 279–292.

[11] I. Schiopu‐Kratina , “Asymptotic Results for GEE With Data From Complex Surveys,” Revue Roumaine de Mathématiques Pures et Appliquées 48, no. 3 (2003): 327–342.

[12] J. C. Deville and C. E. Särndal , “Calibration Estimators in Survey Sampling,” Journal of the American Statistical Association 87, no. 418 (1992): 376–382.

[13] T. Lumley , “Survey: Analysis of Complex Survey Samples,” 2024 R Package Version 4.4.

[14] R Core Team , R: A Language and Environment for Statistical Computing (R Foundation for Statistical Computing, 2024).

[15] B. I. Graubard and T. R. Fears , “Standard Errors for Attributable Risk for Simple and Complex Sample Designs,” Biometrics 61, no. 3 (2005): 847–855. 16135037 10.1111/j.1541-0420.2005.00355.x

[16] P. S. Kott , “The Delete‐a‐Group Jackknife,” Journal of Official Statistics 17, no. 4 (2001): 521.

[17] H. Hartley and J. Rao , “Sampling With Unequal Probabilities and Without Replacement,” Annals of Mathematical Statistics 33, no. 2 (1962): 350–374.

[18] M. E. Thompson and C. Wu , “Simulation‐Based Randomized Systematic PPS Sampling Under Substitution of Units,” Survey Methodology 34, no. 1 (2008): 3.

[19] J. K. Kim and M. Riddles , “Some Theory for Propensity‐Score‐Adjustment Estimators in Survey Sampling,” Survey Methodology 38, no. 2 (2012): 157–165.

[20] N. Carnide , H. Lee , M. R. Frone , A. D. Furlan , and P. M. Smith , “Patterns and Correlates of Workplace and Non‐Workplace Cannabis Use Among Canadian Workers Before the Legalization of Non‐Medical Cannabis,” Drug and Alcohol Dependence 218 (2021): 108386. 33213975 10.1016/j.drugalcdep.2020.108386

[21] “Microdata User Guide, Canadian Tobacco, Alcohol and Drugs Survey, Chapter 10,” 2017, Statistics Canada.

[22] “Methodology of the Canadian Labour Force Survey. Chapter 7,” 2017, Statistics Canada.

[23] N. Carnide , V. Landsman , H. Lee , M. R. Frone , A. D. Furlan , and P. M. Smith , “Workplace and Non‐Workplace Cannabis Use and the Risk of Workplace Injury: Findings From a Longitudinal Study of Canadian Workers,” Canadian Journal of Public Health 114, no. 6 (2023): 947–955. 37523062 10.17269/s41997-023-00795-0 PMC10661545

